# Association Between Implant Positioning Parameters and Prosthetic Failure in Full-Arch Fixed Implant-Supported Prostheses: A Retrospective Matched Case-Control Study

**DOI:** 10.7759/cureus.110180

**Published:** 2026-06-03

**Authors:** Sumanjit Midha, Sami Faisal Jamdar, Sunaina Singla, Jasvinder Kaur, Jeevani Eppala, Jay Gohil, Akriti Mahajan

**Affiliations:** 1 Department of Prosthodontics and Crown and Bridge, Gian Sagar Dental College and Hospital, Rajpura, IND; 2 Department of Oral and Maxillofacial Surgery, Ministry of Health, Riyadh, SAU; 3 Department of Oral and Maxillofacial Surgery, Guru Nanak Dev Dental College and Research Institute, Sunam, IND; 4 Department of Prosthodontics and Crown and Bridge, Guru Nanak Dev Dental College and Research Institute, Sunam, IND; 5 Department of Conservative Dentistry and Endodontics, Nimra Institute of Dental Sciences, Guntur, IND; 6 Department of Prosthodontics and Crown and Bridge, K.M. Shah Dental College and Hospital, Sumandeep Vidyapeeth (Deemed to be University), Vadodara, IND; 7 Department of Oral Medicine and Radiology, Desh Bhagat Dental College and Hospital, Mandi Gobindgarh, IND

**Keywords:** biomechanics, cone-beam computed tomography, implants, prosthesis failure, prosthodontics

## Abstract

Introduction: Successful full-arch rehabilitation depends not only on implant survival but also on long-term prosthetic stability and biomechanical harmony. Despite advancements in implant dentistry, prosthetic complications remain a significant challenge in full-arch fixed implant-supported prostheses. Implant positioning parameters, such as implant angulation, anteroposterior spread, and cantilever extension, may influence the stress distribution and contribute to prosthetic failure. This study aimed to evaluate the association between implant positioning parameters and prosthetic failure in full-arch fixed implant-supported prostheses.

Materials and methods: This retrospective matched case-control study was conducted in the Department of Prosthodontics and Crown and Bridge, using archived patient records from January 2020 to December 2024. Ninety participants were included, comprising 30 patients with prosthetic failure and 60 matched controls without complications. Cone-beam computed tomography (CBCT) records were evaluated to assess the mesiodistal angulation, buccolingual angulation, vertical implant position, anteroposterior spread, distal cantilever length, cantilever-to-anteroposterior spread ratio, and inter-implant distance. Statistical analysis was performed, with a p-value less than 0.05, which was considered statistically significant.

Results: Repeated screw loosening was the most common prosthetic complication and was observed in 13 (43.3%) patients, followed by framework fracture in 11 (36.7%) patients and catastrophic veneering material fracture in 6 (20.0%) patients. The case group demonstrated significantly greater mesiodistal angulation (18.4 ± 4.2° versus 12.1 ± 3.8°), buccolingual angulation (15.7 ± 5.1° versus 10.3 ± 3.6°), vertical implant position (3.8 ± 1.4 mm versus 2.9 ± 0.9 mm), distal cantilever length (14.2 ± 3.1 mm versus 10.4 ± 2.6 mm), and cantilever-to-anteroposterior spread ratio (1.42 ± 0.31 versus 0.89 ± 0.22) compared with controls (p < 0.001). Greater anteroposterior spread and inter-implant distance demonstrated protective effects against prosthetic failure.

Conclusion: Implant positioning parameters significantly influence prosthetic outcomes in full-arch fixed implant-supported prostheses. Increased implant angulation and cantilever extension are associated with a higher prosthetic failure risk, whereas greater anteroposterior spread and inter-implant distance improve biomechanical stability. Accurate prosthetically driven implant placement may reduce technical complications and improve the long-term prosthetic success.

## Introduction

Dental implants have become a widely accepted treatment modality for the rehabilitation of completely edentulous patients requiring full-arch fixed prosthetic restorations. Advances in implant design, surgical protocols, and prosthetic materials have significantly improved the long-term survival and functional outcomes of implant-supported prostheses [1]. Despite the high survival rates, prosthetic complications remain a major clinical concern, particularly in patients with systemic diseases, and may negatively influence patient satisfaction, treatment longevity, maintenance costs, and overall quality of life [2]. Mechanical and technical complications such as framework fracture, repeated screw loosening, and veneering material fracture continue to be reported even in successfully osseointegrated dental implants [3].

Several factors have been associated with prosthetic complications in full-arch fixed implant-supported prostheses, including age, peri-implantitis, lack of osseointegration, occlusal overload, prosthetic design, cantilever extension, implant distribution, and implant angulation [3-5]. Among these variables, implant positioning plays a critical role in achieving favorable biomechanical stress distribution and prosthetic stability [6]. Inaccurate implant positioning may generate excessive forces on the prosthetic framework and supporting components, thereby increasing the likelihood of mechanical failure. Parameters such as mesiodistal angulation, buccolingual angulation, vertical implant position, anteroposterior spread, inter-implant distance, and cantilever-to-anteroposterior spread ratio are considered important determinants of prosthetic success [7].

Although previous studies have evaluated prosthetic complications associated with full-arch fixed implant-supported prostheses, limited evidence is available regarding the direct association between implant positioning parameters and prosthetic failure using a matched case-control methodology. Furthermore, long-term retrospective analyses integrating radiographic measurements with clinical prosthetic outcomes are limited. Therefore, this study aimed to evaluate the association between implant positioning parameters and prosthetic failure in full-arch fixed implant-supported prostheses. The objectives of this study were to assess implant angulation, vertical implant position, anteroposterior spread, cantilever length, cantilever-to-anteroposterior spread ratio, and inter-implant distance using cone-beam computed tomography (CBCT) records and to determine their relationship with prosthetic failure in full-arch fixed implant-supported prostheses.

## Materials and methods

Study design and study setting

The present study was designed as a retrospective matched case-control study conducted in the Department of Prosthodontics and Crown and Bridge at Guru Nanak Dev Dental College and Research Institute, Sunam. The study was conducted between January 2025 and March 2025 using archived patient records from the previous five years, ranging from January 2020 to December 2024. The clinical, prosthetic, and radiographic records of patients rehabilitated with full-arch fixed implant-supported prostheses were retrieved from the institutional database. Ethical approval was granted by the Institutional Ethical Committee of Guru Nanak Dev Dental College, Sunam (GNDDC/IEC/2025/JS45 ). Patient confidentiality and anonymity were maintained throughout the study.

Sample size estimation

Sample size estimation was performed using G*Power statistical software version 3.1.9.7 (Heinrich Heine University Düsseldorf, Düsseldorf, Germany). The calculation was based on a moderate effect size of 0.62 derived from the previously published literature [8]. A statistical power of 80% and a confidence interval of 95% were considered for sample size determination. Using a case-to-control ratio of 1:2, the minimum required sample size was calculated as 90 participants, comprising 30 cases of prosthetic failure and 60 matched controls without prosthetic complications.

Study population and group allocation

The study population consisted of patients rehabilitated with full-arch fixed implant-supported prostheses in either the maxillary or mandibular arch. Archived patient files were screened systematically according to predefined inclusion and exclusion criteria. Only records with complete clinical documentation, prosthetic details, and radiographic findings were included in the analysis.

The case group consisted of 30 patients who presented with definitive prosthetic failure after rehabilitation using full-arch fixed implant-supported prostheses. Prosthetic failure was defined as the occurrence of at least one of the following complications: framework fracture, repeated screw loosening occurring more than twice annually, or catastrophic veneering material fracture requiring complete prosthesis replacement. Only patients with complete clinical and radiographic documentation were included in the case group. The control group consisted of 60 patients rehabilitated with full-arch fixed implant-supported prostheses without any recorded prosthetic complications for a minimum duration of three years after prosthesis loading. Controls were matched with cases at a ratio of 2:1 according to age (within ±5 years), rehabilitated arch, and loading protocol.

Inclusion and exclusion criteria

The inclusion criteria for the study were patients aged ≥18 years, rehabilitation with full-arch fixed implant-supported prostheses, availability of cone-beam computed tomography records in Digital Imaging and Communications in Medicine format, complete clinical and prosthetic documentation, and a minimum follow-up duration of three years after prosthesis delivery. The exclusion criteria were incomplete clinical or radiographic records, rehabilitation with removable implant-supported prostheses, history of major maxillofacial trauma or reconstructive surgery, prosthetic complications associated exclusively with peri-implant infection, and poor-quality cone-beam computed tomography scans unsuitable for radiographic measurements.

Radiographic assessment and implant positioning analysis

Preoperative and postoperative cone-beam computed tomography scans in Digital Imaging and Communications in Medicine format were retrieved from institutional archives. Radiographic analysis was performed by a blinded and calibrated examiner using Carestream Dental Imaging Software version 8.0 (Carestream Dental Ltd., Atlanta, GA). The evaluated implant positioning parameters included mesiodistal implant angulation relative to the prosthetic axis, buccolingual implant angulation relative to the occlusal reference plane, vertical implant position measured as the distance between the implant platform and the planned occlusal plane, anteroposterior spread measured as the linear distance between the anterior-most and posterior-most dental implants within the rehabilitated arch, distal cantilever length, cantilever-to-anteroposterior spread ratio, and inter-implant distance between adjacent dental implants. All measurements were recorded in degrees (°) or millimeters (mm), as applicable (Table 1).

**Table 1:** Implant positioning parameters: definitions, measurement methods, and units AP: anteroposterior, CBCT: cone‑beam computed tomography

Parameter	Definition	Measurement method	Units/notes
Implant angulation	Mesiodistal and buccolingual angulation of each implant relative to a reference vertical axis (long axis of adjacent implant or line perpendicular to occlusal plane); maximum pairwise angular discrepancy between any two implants also recorded	Cross-sectional CBCT slices through implant center; angle measurement tool in implant planning software (Carestream Dental Imaging Software version 8.0).	Degrees (°), absolute deviation from 0° (parallelism)
Vertical implant position	Distance from implant platform (most coronal aspect) to crestal bone level; positive = supragingival (above bone), negative = subcrestal; also expressed relative to prosthetic finish line	Mesial and distal aspects on reformatted CBCT sections	Millimeters (mm)
AP spread	Horizontal distance parallel to occlusal plane between most anterior implant platform center and most posterior implant platform center (same side for unilateral; for full-arch: anterior to midpoint of line between two most posterior implants)	Axial CBCT views	Millimeters (mm)
Cantilever length	Distance from distal edge of most posterior implant platform to distal extension of prosthesis framework; for anterior cantilevers: from most anterior implant platform to incisal edge	Definitive prosthesis aligned with implant positions	Millimeters (mm)
Cantilever-to-AP spread ratio	Cantilever length divided by anteroposterior spread	Calculated from measured values; for full-arch prostheses, larger cantilever length of the two hemiarches is used	Dimensionless ratio
Inter-implant distance	Center-to-center distance between adjacent implants at implant platform level; shortest surface-to-surface distance at bone crest also recorded for non-parallel implants	Axial CBCT data	Millimeters (mm); closely spaced defined as center-to-center <7 mm

Examiner calibration and reliability analysis

To evaluate intra-examiner reliability, 20% of the cone-beam computed tomography scans were reassessed after a two-week interval by the same examiner under identical conditions. Intraclass correlation coefficient analysis was performed to determine the reproducibility and reliability of radiographic measurements.

Outcome measures

The primary outcome measure in this study was prosthetic failure in the full-arch fixed implant-supported prostheses. The secondary outcome measures included the evaluation of specific implant positioning parameters such as implant angulation, vertical implant position, anteroposterior spread, cantilever length, cantilever-to-anteroposterior spread ratio, and inter-implant distance and their association with mechanical and prosthetic complications, including screw loosening, framework fracture, and veneering material fracture in full-arch fixed implant-supported prostheses (Table 1).

Statistical analysis

Statistical analysis was performed using Statistical Package for the Social Sciences software (version 26.0; IBM Corp., Armonk, NY). Continuous variables are expressed as mean and standard deviation (SD), whereas categorical variables are presented as frequencies and percentages. Normality of data distribution was assessed using the Shapiro-Wilk test. An independent-samples t-test was used for normally distributed continuous variables, whereas the Mann-Whitney U test was applied for non-normally distributed variables. The chi-squared test was used for categorical variables. Conditional logistic regression analysis was performed to determine the predictors of prosthetic failure and calculate odds ratios with 95% confidence intervals (CIs). Statistical significance was set at p < 0.05.

## Results

Table 2 presents the demographic and clinical characteristics of study participants. The mean age of patients in the case group was 54.3 ± 8.7 years, whereas the control group showed a mean age of 53.8 ± 9.1 years, with no statistically significant intergroup difference (p = 0.803). Male participants constituted 16 (53.3%) in the case group and 38 (63.3%) in the control group, whereas female participants accounted for 14 (46.7%) and 22 (36.7%) participants, respectively. Immediate loading was observed in 12 (40.0%) and 21 (35.0%) controls, whereas delayed loading was reported in 18 (60.0%) and 39 (65.0%) controls. No statistically significant differences were observed between the groups regarding follow-up duration, number of dental implants per arch, implant diameter, implant length, bone quality, or prosthetic material used (p > 0.05), indicating comparability between the groups.

**Table 2:** Comparison of demographic and clinical characteristics between cases and controls Data presented as mean ± SD and frequency (percentage) p-value less than 0.05 considered statistically significant χ² = Chi-square test t = Independent samples t-test SD: standard deviation

Characteristic	Categories	Cases (n = 30)	Controls (n = 60)	Test value	p-value
Age (years)	mean ± SD	54.3 ± 8.7	53.8 ± 9.1	t = 0.25	0.803
Sex, number (%)	Male	16 (53.3)	38 (63.3)	χ² = 0.83	0.361
	Female	14 (46.7)	22 (36.7)		
Implant procedure, number (%)	Immediate	12 (40.0)	21 (35.0)	χ² = 0.21	0.643
	Delayed	18 (60.0)	39 (65.0)		
Implant characteristic	Follow-up duration (months), mean ± SD	42.4 ± 14.2	41.8 ± 18.6	t = -1.26	0.118
	Number of implants per arch, mean ± SD	5.6 ± 0.7	5.8 ± 0.8	t = -1.21	0.214
	Implant diameter (mm), mean ± SD	4.1 ± 0.5	4.2 ± 0.4	t = -0.95	0.312
	Implant length (mm), mean ± SD	11.3 ± 1.6	11.6 ± 1.4	t = -0.87	0.368
Bone quality, number (%)	Type I/II	21 (70.0)	44 (73.3)	χ² = 0.11	0.732
	Type III/IV	9 (30.0)	16 (26.7)		
Prosthetic material, number (%)	Zirconia	18 (60.0)	37 (61.7)	χ² = 0.02	0.879
	Porcelain fused metal	12 (40.0)	23 (38.3)		

Table 3 shows the distribution of prosthetic complications between the groups. Repeated screw loosening was the most common complication, reported in 13 (43.3%) patients, followed by framework fracture in 11 (36.7%) patients and catastrophic veneering material fracture in 6 (20.0%) patients. The median onset time was 18.5 months for repeated screw loosening, 34.0 months for framework fracture, and 28.0 months for catastrophic veneering material fracture. The overall median time to prosthetic complication was 24.0 months after prosthesis delivery.

**Table 3:** Distribution and onset of prosthetic complications in the case group Data presented as frequency (percentage) and median (IQR) Onset duration defined as the time interval between prosthesis delivery and the first recorded complication event Percentages calculated based on the total number of cases IQR: interquartile range

Complication type	Number (%)	Onset in months, median (IQR)
Framework fracture	11 (36.7)	34.0 (22-51)
Repeated screw loosening (>2 episodes/year)	13 (43.3)	18.5 (11-29)
Catastrophic veneering material fracture	6 (20.0)	28.0 (17-42)
Total	30 (100.0)	24.0 (14-39)

Table 4 compares the implant positioning parameters between the case and control groups. The case group demonstrated significantly greater mesiodistal angulation (18.4 ± 4.2°) and buccolingual angulation (15.7 ± 5.1°) than the control group (12.1 ± 3.8° and 10.3 ± 3.6°, respectively) (p < 0.001). The vertical implant position was significantly higher in the case group (3.8 ± 1.4 mm) than in the control group (2.9 ± 0.9 mm) (p = 0.001). The control group showed significantly greater anteroposterior spread, whereas the distal cantilever length and cantilever-to-anteroposterior spread ratio were significantly higher in the case group (p < 0.001). The inter-implant distance was significantly lower in the case group than in the control group (p < 0.001).

**Table 4:** Comparison of implant positioning parameters between cases and controls Data presented as mean ± SD p-value less than 0.05 considered statistically significant * indicates statistically significant difference U = Mann-Whitney U test t = Independent samples t-test AP: distance between the most anterior implant to most posterior implant, SD: standard deviation

Positioning parameter	Cases (n = 30), mean ± SD	Controls (n = 60), mean ± SD	Test statistic	p-value
Mesiodistal angulation (°)	18.4 ± 4.2	12.1 ± 3.8	t = 7.43	<0.001*
Buccolingual angulation (°)	15.7 ± 5.1	10.3 ± 3.6	t = 5.91	<0.001*
Vertical implant position (mm)	3.8 ± 1.4	2.9 ± 0.9	U = 586	0.001*
AP spread (mm)	19.3 ± 3.7	23.6 ± 4.1	t = -5.27	<0.001*
Distal cantilever length (mm)	14.2 ± 3.1	10.4 ± 2.6	t = 6.39	<0.001*
Cantilever-to-AP spread ratio	1.4 ± 0.3	0.9 ± 0.2	U = 234	<0.001*
Inter-implant distance (mm)	8.1 ± 1.4	9.3 ± 1.6	t = -3.81	<0.001*

Table 5 presents the conditional logistic regression analysis of predictors of prosthetic failure. Increased mesiodistal angulation, buccolingual angulation, vertical implant position, distal cantilever length, and cantilever-to-anteroposterior spread ratio were significantly associated with an increased risk of prosthetic failure (p < 0.05). Among these variables, the cantilever-to-anteroposterior spread ratio demonstrated the strongest association with prosthetic failure, with an odds ratio of 8.94. Conversely, greater anteroposterior spread and increased inter-implant distance were identified as protective factors against prosthetic failure.

**Table 5:** Conditional logistic regression analysis for predictors of prosthetic failure Conditional logistic regression analysis performed using matched case-control sets with a case-to-control ratio of 1:2 Data presented as regression coefficient, odds ratio, and 95% CI with lower and upper limits All variables entered simultaneously using the enter method p-value less than 0.05 considered statistically significant * indicates statistically significant association χ² = Wald chi-square value AP: distance between the most anterior implant to most posterior implant, CI: confidence interval, OR: odds ratio

Variable	Regression coefficient (β)	OR	95% CI	Wald χ²	p-value
Mesiodistal angulation (°)	+0.39	1.48	1.21-1.81	14.87	<0.001*
Buccolingual angulation (°)	+0.27	1.31	1.09-1.57	8.42	0.004*
Vertical implant position (mm)	+0.54	1.72	1.19-2.49	8.17	0.004*
AP spread (mm)	-0.33	0.72	0.61-0.85	14.23	<0.001*
Distal cantilever length (mm)	+0.61	1.84	1.38-2.46	17.09	<0.001*
Cantilever-to-AP spread ratio	+2.19	8.94	3.47-23.01	22.64	<0.001*
Inter-implant distance (mm)	-0.44	0.64	0.48-0.86	9.36	0.002*

## Discussion

This retrospective matched case-control study evaluated the association between implant positioning parameters and prosthetic failure in full-arch fixed implant-supported prostheses. The findings demonstrated that increased implant angulation, greater vertical implant position, increased distal cantilever length, and higher cantilever-to-anteroposterior spread ratio were significantly associated with prosthetic failure, whereas greater anteroposterior spread and inter-implant distance appeared to provide a protective effect. These findings emphasize the importance of prosthetically driven implant placement and biomechanical considerations during full-arch rehabilitation.

Demographic and clinical variables did not show statistically significant differences between the case and control groups, indicating that both groups were adequately matched. Similar observations were reported by Papaspyridakos et al. [9], who demonstrated that prosthetic complications in full-arch fixed implant-supported prostheses were more strongly influenced by prosthetic and biomechanical factors than by patient demographic variables alone. In contrast, a retrospective clinical study by Sabău et al. [10] evaluated the mechanical and biological complications occurring two years after full-arch implant-supported prosthetic rehabilitation in edentulous patients. The authors found that both complication types were relatively common, with patient-related factors, such as younger age and absence of systemic disease, being more strongly associated with complications than prosthetic design variables.

Among the prosthetic complications observed in this study, repeated screw loosening was the most frequent complication. This finding corresponds to a previous study that identified screw loosening as one of the most commonly reported technical complications associated with full-arch fixed implant-supported prostheses [8]. Screw loosening may result from excessive occlusal loading, implant misalignment, poor passive fit of the framework, or cantilever-related stress. Increased mechanical stress at the implant-abutment interface may progressively reduce screw preload and contribute to prosthetic instability over time [11].

This study demonstrated significantly greater mesiodistal and buccolingual angulations in the prosthetic failure group. Excessive implant angulation may compromise axial load distribution, increase bending moments on the prosthetic framework, and support dental implants [12]. A finite element study conducted by Sertgöz and Güvener [13] demonstrated that non-parallel implant positioning produces higher stress concentrations around implant components and prosthetic frameworks. Similarly, Rangert et al. [14] reported that unfavorable implant angulation contributes to increased biomechanical overload and technical complications in implant-supported prostheses. These findings support the present results and indicate that improper implant angulation may predispose prostheses to framework fractures, veneering material fractures, or screw loosening.

The vertical implant position was significantly greater in the prosthetic failure group. An increased vertical distance between the implant platform and occlusal plane may increase the crown-to-implant ratio and magnify leverage forces during mastication. Blanes et al. [15] reported that increased crown-to-implant ratios were associated with greater biomechanical stress and prosthetic complications in implant-supported restorations. Therefore, excessive prosthetic height may contribute to higher mechanical loading and long-term technical failure.

The present study further demonstrated that the distal cantilever length and cantilever-to-anteroposterior spread ratio were significantly associated with prosthetic failure, whereas a greater anteroposterior spread demonstrated a protective effect [16]. These findings are consistent with the biomechanical principles described by Skalak [17], who emphasized that longer cantilever extensions significantly increase the bending forces on implant-supported prostheses. Krekmanov et al. [18] recommended tilting implants in the posterior region for better prosthesis support. The strong predictive value of the cantilever-to-anteroposterior spread ratio observed in the present study indicated that minimizing cantilever extension is critical for long-term prosthetic stability.

The inter-implant distance was significantly lower in the prosthetic failure group than in the control group. Reduced spacing between adjacent dental implants may limit favorable stress distribution and compromise the framework support. Chang and Wennström [19] evaluated peri-implant bone alterations around implant-supported fixed dental prostheses in relation to inter-unit distances. The authors reported that shorter inter-unit distances were associated with greater marginal bone loss, suggesting that prosthetic design and spacing between implant units may influence the long-term peri-implant bone stability. Therefore, proper implant spacing and distribution are essential to achieve biomechanical stability in full-arch fixed implant-supported prostheses.

The clinical implications of this study highlight the importance of precise implant positioning and comprehensive prosthetic planning in full-arch rehabilitation. Appropriate implant angulation, adequate inter-implant distance, wider anteroposterior spread, and minimized cantilever extension may reduce mechanical complications and improve long-term prosthetic outcomes. The use of cone-beam computed tomography-guided surgical planning and digitally guided implant placement protocols may further improve the implant positioning accuracy and biomechanical performance.

Despite these significant findings, the present study had several limitations. The retrospective study design may have introduced selection bias and dependence on previously recorded patient data. This study was limited to records obtained from a single institutional setting and associated centers, which may affect the generalizability of the findings. Although radiographic assessment protocols were standardized, complete standardization of prosthetic and occlusal variables, including occlusal scheme, opposing dentition, prosthesis design characteristics, and clinician-related restorative factors, was not feasible because of the retrospective multiclinical record-based design. In addition, precise assessment of prosthetic dimensions, such as cantilever extension and occlusal reference plane, was limited by the retrospective reliance on archived CBCT datasets without standardized digital prosthesis superimposition workflows or universally available STL records. These variables may have influenced prosthetic outcomes and should be considered in future prospective investigations. Future prospective multicenter studies with larger sample sizes, standardized digital workflows, and longer follow-up durations are recommended to validate the relationship between implant positioning parameters and prosthetic failure in full-arch fixed implant-supported prostheses.

## Conclusions

Within the limitations of the present retrospective matched case-control study, implant positioning parameters demonstrated a significant association with prosthetic failure in full-arch fixed implant-supported prostheses. Increased implant angulation, greater vertical implant position, longer distal cantilever length, and higher cantilever-to-anteroposterior spread ratio were associated with an increased risk of prosthetic complications, whereas greater anteroposterior spread and inter-implant distance showed protective effects. Accurate prosthetically driven implant placement and careful biomechanical planning are essential for minimizing technical complications and improving the long-term success of full-arch fixed implant-supported prostheses.
